# Predict the Future Incidence and Mortality of Breast Cancer in Iran from 2012–2035

**Published:** 2017-04

**Authors:** Ali Asghar VALIPOUR, Mahdi MOHAMMADIAN, Mahin GHAFARI, Abdollah MOHAMMADIAN-HAFSHEJANI

**Affiliations:** 1. Dept. of Public Health, School of Public Health, Abadan School of Medical Sciences, Abadan, Iran; 2. Dept. of Social Medicine, School of Medicine, Dezful University of Medical Sciences, Dezful, Iran; 3. Dept. of Public Health, School of Public Health, Shahrekord University of Medical Sciences, Shahrekord, Iran; 4. Dept. of Epidemiology and Biostatistics, Isfahan University of Medical Sciences, Isfahan, Iran; 5. Dept. of Epidemiology and Biostatistics, School of Public Health, Tehran University of Medical Sciences, Tehran, Iran

## Dear Editor-in-Chief

Global Breast Cancer (BC) is the most common non-skin malignancy, nearly a third of newly diagnosed cancers in the United States and the second leading cause of mortality in women throughout the world was BC (1, 2). Between 1975 and 2000 the burden of BC has doubled, that is attributable to the increase in life expectancy and spread of unhealthy lifestyle (3). Nevertheless, these trends are not visible in early onset of BC, as the rates have been more or less stable in most countries in the past 20 yr (4). As for mortality rates, they have been progressively decreasing, particularly in younger women, due to the improved treatment and primary detection (5). In Iran with increasing life expectancy and the aging of the residents, the incidence and mortality of BC will increase in the future years (6).

**Fig. 1:**
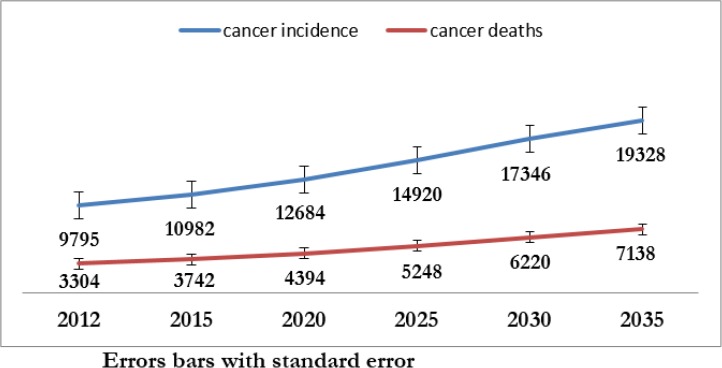
Predict the number of incidence and mortality of breast cancers in Iran

In GLOBOCAN project, the expected number of new cancer cases or deaths in a country or region in 2015–2035 is computed by multiplying the age-specific incidence/mortality rates estimated for 2012, by the corresponding expected population for 2015–2035. In Iran, based on the GLOBOCAN project in 2012, the number of BC in woman was 9795 case and the number of new case have an increase in the next few years, so in 2015, 2020, 2025, 2030 and 2035 the number of new cases were 10982, 12684, 14920, 17346 and 19328, respectively. Therefore, in 2035 compare 2012 the numbers of new case were nearly 2 times. In addition, in 2012, the numbers of death from BC was 3304 and in the next few years, we have increase in the number of death from BC, so in 2015, 2020, 2025, 2030 and 2035 the numbers of deaths were 3742, 4394, 5248, 6220 and 7138. We expect that Iranian population structure, the greatest increase in the number of new cases and deaths from BC observe in age group (ages >= 65), so in 2035 compared to 2012, the number of new case and mortality will be 3 times, while in the age group below 65 yr, the increase is about 1.8 times (7) (Table 1, Fig. 1).

**Table 1:** Predict the number of incidence and mortality from breast cancers in Iran

**Year**	**Estimated number of new cancers**			**Estimated number of cancer deaths**		
	**Ages < 65**	**Ages >= 65**	**All**	**Ages < 65**	**Ages >= 65**	**All**
2012	8390	1405	9795	2465	839	3304
2015	9342	1640	10982	2772	970	3742
2020	10556	2128	12684	3154	1240	4394
2025	12203	2717	14920	3661	1587	5248
2030	13886	3460	17346	4184	2036	6220
2035	14985	4343	19328	4565	2573	7138

Population forecasts were extracted from the United Nations, World Population prospects, the 2012 revision.

Numbers are computed using age-specific rates and corresponding populations for 10 age groups. GLOBOCAN 2012 (IARC) - 15.3.2016

We predicted the number of incidence and mortality of BC in Iran between 2012 and 2035. Although these results should be taken with caution given that they were obtained using prediction methods, it is important to highlight that there is a trend for increased incidence and mortality of BC in Iran. For this reason, in addition to planning for prevention of disease occurrence in the population at risk, the health policy makers should anticipate and provide diagnostic tools, therapeutic facilities, and skilled healthcare workers to cope with these additional patients in the next few years.
